# The role of aspartic acid in reducing coral calcification under ocean acidification conditions

**DOI:** 10.1038/s41598-020-69556-0

**Published:** 2020-07-30

**Authors:** Celeste Kellock, Catherine Cole, Kirsty Penkman, David Evans, Roland Kröger, Chris Hintz, Ken Hintz, Adrian Finch, Nicola Allison

**Affiliations:** 1 School of Earth and Environmental Sciences, University of St. Andrews, St. Andrews, KY16 9AL UK; 2 Department of Chemistry, University of York, York, UK; 3 Department of Physics, University of York, York, UK; 4 Department of Marine and Environmental Sciences, Savannah State University, Savannah, GA USA; 5 Department of Electrical and Computer Engineering, George Mason University, Fairfax, VA USA; 6 Present Address: Biological and Environmental Sciences, School of Natural Sciences, University of Stirling, Stirling, FK9 4LA Scotland, UK; 7 Present Address: Centre of Science Communication, University of Otago, Dunedin, 9016 New Zealand; 8 Present Address: Institute of Geosciences, Goethe University Frankfurt, Frankfurt am Main, Germany

**Keywords:** Marine biology, Climate-change impacts

## Abstract

Biomolecules play key roles in regulating the precipitation of CaCO_3_ biominerals but their response to ocean acidification is poorly understood. We analysed the skeletal intracrystalline amino acids of massive, tropical *Porites* spp. corals cultured over different seawater pCO_2_. We find that concentrations of total amino acids, aspartic acid/asparagine (Asx), glutamic acid/glutamine and alanine are positively correlated with seawater pCO_2_ and inversely correlated with seawater pH. Almost all variance in calcification rates between corals can be explained by changes in the skeletal total amino acid, Asx, serine and alanine concentrations combined with the calcification media pH (a likely indicator of the dissolved inorganic carbon available to support calcification). We show that aspartic acid inhibits aragonite precipitation from seawater in vitro, at the pH, saturation state and approximate aspartic acid concentrations inferred to occur at the coral calcification site. Reducing seawater saturation state and increasing [aspartic acid], as occurs in some corals at high pCO_2_, both serve to increase the degree of inhibition, indicating that biomolecules may contribute to reduced coral calcification rates under ocean acidification.

## Introduction

Tropical coral skeletons are major components of coral reefs and provide substrates and habitat spaces for fisheries and protection from wave erosion for coastal communities^1^. Ocean acidification, caused by rising seawater pCO_2_, typically suppresses the calcification rates of tropical corals and threatens the formation of these structures^2^. Coral skeletons are formed from semi-isolated extracellular calcification media (ECM) contained between the base of the coral tissues and the underlying skeletons^3^. Ocean acidification reduces the pH of this ECM (termed pH_ECM_), shifts the dissolved inorganic carbon (DIC) equilibrium to the detriment of CO_3_^2−^ and likely reduces the ECM saturation state (Ω), a measure of both the [CO_3_^2−^] and [Ca^2+^] available for mineral precipitation^4^. Coral skeletons are composite materials, composed of aragonite (a calcium carbonate polymorph) and organic macromolecules which are disseminated in the mineral phase at the nanoscale^5^. This intracrystalline skeletal organic matrix (SOM) include proteins, sugars, polysaccharides and lipids and is implicated in the control of mineral precipitation^6^. For example, primary coral cell cultures produce extracellular organic materials on which aragonite precipitates^7^ while SOM extracted from tropical, Mediterranean and deep sea coral skeletons affects the precipitation rate, morphology and polymorphism of CaCO_3_ precipitated in vitro^8–10^. Organic fibrils at the coral calcification site concentrate Ca^2+^^11^ and several proteins, lipids and macromolecules resolved from coral SOMs are capable of binding Ca^2+^^12,13^. Aspartic acid is typically the most abundant amino acid in the coral SOM^12,14^ and influences multiple stages of CaCO_3_ nucleation and precipitation^15,16^. L-aspartic acid forms complexes with Ca^2+^, probably via the negatively charged carboxylic acid group (COO^−^) on the side chain, and this may provide the mechanism to control CaCO_3_ precipitation and morphology^16^.

Resolving how the organic component of the skeleton responds to increases in seawater pCO_2_ is critical to understand the effects of ocean acidification on coral accretion. Increasing seawater pCO_2_ increases the concentration of skeletal protein observed in coral skeletons^17^ and is inferred to increase skeletal organic content^18^. Changes in pH_ECM_ in response to increasing seawater pCO_2_, may alter the net negative charge of biomolecules and thereby influence their control of CaCO_3_ precipitation^19^. Here we identify large variations in the amino acid compositions of a suite of massive *Porites* spp. corals cultured over a range of seawater pCO_2_^20–22^. We find that amino acid concentrations are significantly correlated with coral calcification rates and we explore how aspartic acid, the most prevalent skeletal amino acid, affects skeleton formation by precipitating aragonite in vitro*,* under fluid conditions analogous to those of the coral calcification site.

## Results and discussion

### Intracrystalline amino acids and coral calcification

**Figure 1:**
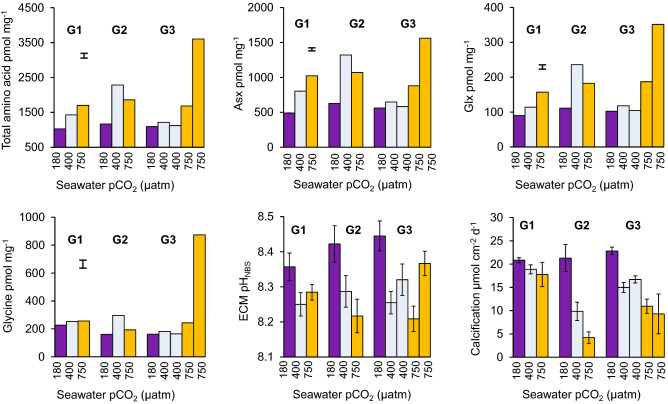
Skeletal amino acid concentrations of each coral genotype (G1, G2 and G3) cultured at 25 °C and over a range of seawater pCO_2_. Two replicate colonies of G3 were cultured at 400 and 750 µatm. Error bars indicate the mean standard deviation of analyses of duplicate drilled samples. Calcification rates^20^ (error bar shows typical standard deviation of 3–4 measurements per colony) and calcification site pH^21^ (adjusted to the pH_NBS_ scale, error bars show 95% confidence limits) are shown for reference. *Asx* aspartic acid + asparagine, *Glx* glutamic acid + glutamine.

**Figure 2:**
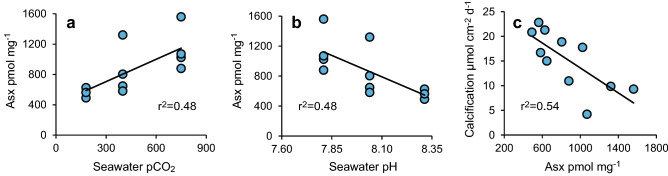
Regressions between skeletal Asx concentration and (**a**) seawater pCO_2_, (**b**) seawater pH and (**c**) coral calcification rate^20^.

We analysed the intracrystalline amino acids of the skeletons of 3 genotypes of massive *Porites* spp. corals cultured at 25 °C and at 3 different seawater pCO_2_ conditions namely 180 µatm, 400 µatm and 750 µatm^20–22^ (Fig. 1, Table S1). Concentrations of total amino acids, aspartic acid/asparagine (termed Asx), glutamic acid/glutamine (termed Glx) and alanine exhibit significant positive correlations with seawater pCO_2_ and inverse correlations with seawater pH (Table S2). Strongest correlations are observed between these seawater parameters and Asx and are illustrated in Fig. 2a, b. No significant correlations were observed between concentrations of any skeletal amino acids and either pH_ECM_ or [H^+^]_ECM_.

**Figure 3:**
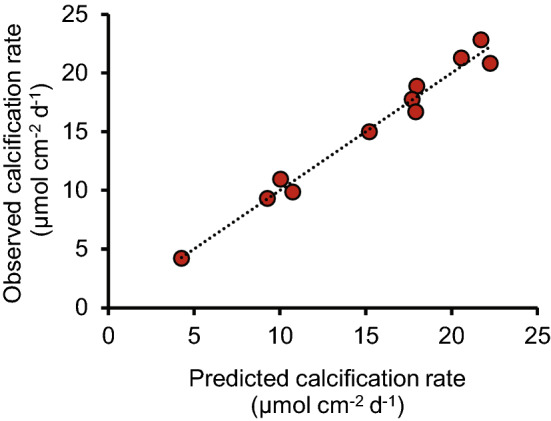
Observed and predicted coral calcification rates. Predicted rates were modelled using a multiple linear regression model of concentrations of Asx, Glx, serine, alanine and total amino acid and pH_ECM_.

Significant inverse linear correlations occur between coral calcification rate (reported previously^20^) and skeletal Asx (Fig. 2c), serine, alanine, Glx and total amino acids (Figure S1, Table S2). Multiple linear regression analysis indicate that a high degree of variance in calcification rate (the dependent variable) is correlated with these amino acid concentrations (the independent variables, *p* = 0.016, r^2^ = 0.90, see Table S3 for individual parameters). For comparison r^2^ = 0.57–0.60 for regressions between coral calcification rate and seawater pCO_2_ and seawater pH. Precipitation rates of inorganic aragonites are highly dependent on the seawater saturation state, Ω (reflecting the availability of CO_3_^2−^ for incorporation in the CaCO_3_ precipitate)^23^. The [CO_3_^2^] of the calcification fluid is a function of total DIC and pH. Adding the pH_ECM_ for these corals (inferred from δ^11^B analysis of the coral skeletons^21^) into the regression increases both the significance and coefficient of determination (*p* = 0.0028, r^2^ = 0.98, Table S3, Fig. 3). We cultured and analysed two duplicate colonies of the *P. murrayensis* genotype at 400 and 750 μatm seawater pCO_2_ and observed large variations in skeletal amino acid concentrations between one pair of duplicates (G3, 750 µatm, Fig. 1). We also observe large variations in pH_ECM_ between these corals^21^ (Fig. 1) but we are able to explain almost all of the variation in coral calcification between them (and the other colonies) on the basis of skeletal amino acids and pH_ECM_ in combination.

The concentrations of some amino acids are highly correlated over the dataset e.g. for a linear regression of [Asx] and [Glx] r^2^ = 0.90. This multicollinearity does not affect the coefficient of determination of the multiple linear regression model (i.e. describing how well the independent variables predict the dependent variable) but does increase the likelihood that we underestimate the significance of one or more amino acids in the regression model and that we misidentify the amino acid which may be responsible for changes in calcification rate.

It is unclear if higher skeletal [amino acid] reflects variations in the coral production of amino acid or enhanced skeletal incorporation of the produced amino acid, but we do not observe consistent relationships between the total skeletal amino acid produced each day (calculated from skeletal concentration and calcification rate^20^ and seawater pCO_2_, Figure S2a). We do not observe a significant correlation between the total amount of Asx (or other amino acids) produced each day and coral calcification rate (Figure S2b) to support the interpretation that calcification is limited by an energy budget required to synthesise the skeletal organic matrix^21^ (assuming that amino acid concentrations are reflective of the total SOM).

Asparagine (and glutamine) undergo deamination during the amino acid extraction process and we cannot separately quantify aspartic acid and asparagine or glutamic acid and glutamine by this method. Proteomic methods, suggest that aspartic acid is a major component of skeletal proteins^24^ and it is likely that the asparagine contribution to Asx is small. We observe inverse correlations between Asx and both glycine and leucine (Figure S3) which reflect changes in the composition of intracrystalline proteins but the reason for this is unclear.

### Interactions of aspartic acid and aragonite precipitation in vitro

**Figure 4:**
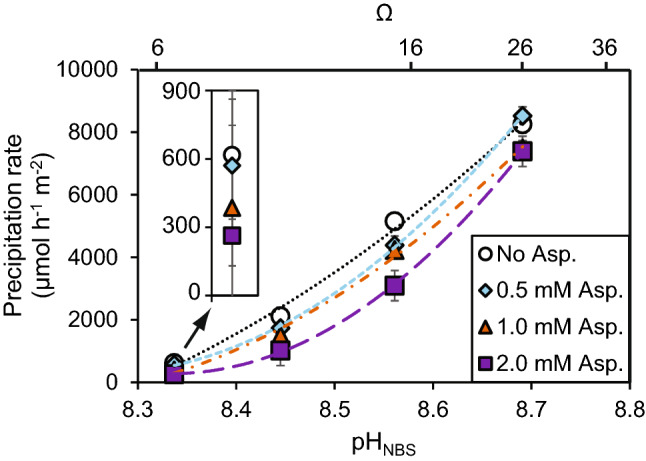
Aragonite precipitation rates from seawater in vitro as a function of [aspartic acid], pH and Ω at seawater pCO_2_ = 400 µatm. The inset shows the points at pH 8.34 on an expanded axis. Error bars indicate standard deviations of replicate precipitations (n = 2–10) and are usually smaller than the symbols.

**Figure 5:**
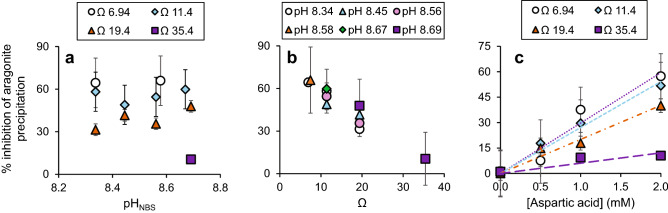
The % inhibition of aragonite precipitation (calculated by comparing mean precipitation with aspartic acid with the mean rate observed with no added aspartic acid) as a function of (**a**) pH at 2 mM aspartic acid and various Ω, (**b**) Ω at 2 mM aspartic acid and at various pH and (**c**) [aspartic acid] and Ω. Error bars were calculated by compounding the standard deviations of precipitations with and without aspartic acid.

The coral skeleton analyses demonstrate that Asx (assumed to be predominantly composed of aspartic acid) is the most prevalent amino acid in the skeletons (Fig. 1) and is the amino acid most strongly correlated with coral calcification rate (Table S2). To explore the potential roles of aspartic acid in coral calcification we precipitated synthetic aragonites from seawater at the approximate pH^21^ and soluble amino acid concentrations (see “Methods” section) inferred to occur at the coral calcification site. In our initial experiments (at seawater pCO_2_ = 400 µatm and with pH and omega (Ω) co-varying) aspartic acid inhibited aragonite precipitation and inhibition was more pronounced at higher [aspartic acid] and at lower pH/Ω (Fig. 4). We conducted a second series of experiments, under varying seawater pCO_2_ with either constant pH (varying Ω) or constant Ω (varying pH) to separate the interactions of pH and Ω with aspartic acid during aragonite precipitation (Fig. 5a, b). A multiple linear regression model of the entire dataset (Table S4) indicates that aragonite precipitation rates are significantly affected by both [aspartic acid] (*p* = 5.1 × 10^–20^, Fig. 5c) and Ω (*p* = 3.2 × 10^–58^, Fig. 5a) but are independent of pH (*p* = 0.40, Fig. 5b). As far as we are aware, decoupling the omega and pH, and finding omega to be the principal driver of aspartic acid induced aragonite precipitation inhibition, is a unique observation.

The interaction of aspartic acid in aragonite precipitation is not fully understood. The precipitation of CaCO_3_ from a solution can occur via multiple stages, and organic additives have the ability to both promote and inhibit different stages^15,16^. In brief, homogenous nucleation of CaCO_3_ occurs in the absence of nucleation sites and likely proceeds via the formation of pre-nucleation clusters of the constituent ions of the solid (or other chemical species) which aggregate and dehydrate to form amorphous solids^25^. Biomolecules can act as templates for the aggregation of these amorphous nanoparticles which then develop into crystal domains after reaching a critical size^26^. Heterogeneous nucleation occurs in the presence of existing nucleation surfaces e.g. a mineral seed or an organic material, and requires the formation and subsequent growth of a nucleus on the existing surface. It is not known if ions or clusters of species are involved in these processes^25,27^. Much of the existing literature on biomolecule interactions during CaCO_3_ precipitation focuses on calcite and vaterite rather than aragonite and there are contradictory reports of biomolecule effects. Aspartic acid accelerated the nucleation of vaterite in seeded^28^ and unseeded^16^ precipitations but stabilised pre-nucleation clusters and delayed CaCO_3_ nucleation in another report^15^, consistent with computations indicating that aspartic acid reduces ion dissolution and aggregation in solution^29^^.^ Interactions of aspartic acid at the solution:solid interface may promote calcite precipitation rate by decreasing the energy barrier to the attachment of solutes^19^, or may inhibit precipitation by blocking subsequent ion attachment^30^. Discrepancies between studies may reflect the impact of varying environmental factors, e.g. ionic strength^31^, pH (affecting molecule charge^19^), biomolecule concentration^19^ and the presence of other cations e.g. Mg^2+^^32^. Acidic proteins (rich in aspartic acid) can bind to and inhibit extension of specific faces of calcite crystals thereby regulating growth and crystal morphology^33^ and also bind Ca^2+^ and catalyse the precipitation of aragonite in vitro^34^.

In this study, aragonite precipitation from seawater is inhibited by aspartic acid at the concentrations inferred to occur in the coral ECM. Calcite propagation rates were also inhibited at similar [aspartic acid] in lower ionic strength solutions^19^. Inhibition of precipitation likely reflects aspartic acid adsorption at the crystal:solution interface which impedes the attachment of ions to further CaCO_3_ growth^30^. This inhibitory effect is less pronounced at higher Ω (Fig. 5a). Aragonite precipitation rates were constant throughout each precipitation (Figure S4), indicating that the surface area for CaCO_3_ growth did not change. Variations in precipitation rate between experiments likely reflect crystal growth (rather than pre-nucleation or nucleation events). A potential explanation for our observations is that the ability of aspartic acid to adsorb to aragonite is reduced at high Ω. Increased CaCO_3_ lattice disorder is inferred in synthetic aragonites precipitated at high seawater Ω in the absence of biomolecules^35^. This may hamper the ability of aspartic acid to adsorb to the solid:solution interface (and inhibit subsequent precipitation). Alternatively, faster aragonite growth (at high Ω) could itself disadvantage the adsorption of aspartic acid to the growing crystal. It is unlikely that variations in Ω in this study (driven by changes in [CO_3_^2−^]) significantly affect aspartic acid speciation. Between pH 8 and 9, aspartic acid speciation in seawater is dominated by Mg(asp)H^+^, Mg(asp)^0^ and (asp)H^-^ with Ca complexes playing a minor role (< 10% of aspartic acid)^36^. We observe no significant impact of pH on aragonite precipitation by aspartic acid (Fig. 5b). This likely reflects the large difference between aspartic acid pKa (~ 1.99, 3.90 and 9.90 at 25 °C) and the pH range studied here (pH_NBS_ 8.34–8.69).

We note that the free amino acid used in the precipitations here is not observed in high concentrations in extracts of coral SOM^37^, where aspartic acid is combined with other amino acids into peptides and proteins. Small aspartic acid-rich peptides inhibit calcite precipitation at lower concentrations than Asp-dipeptides or free aspartic acid^19^ and adsorbing the amino acid to a template in small pore spaces may influence its effect on CaCO_3_ precipitation^38^. However, the interaction of aspartic acid with precipitating CaCO_3_ is likely due to the acidic carboxylic acid side chain which is present in both the free and protein-bound forms of the amino acid. We interpret our data to indicate an increase in aspartic acid (in whatever form) in the coral ECM under ocean acidification conditions and our in vitro precipitation experiments indicate that an increase in ECM [aspartic acid] could suppress coral calcification at high seawater pCO_2_.

### Implications for coral response to ocean acidification

Our experiments provide insights into the potential role of aspartic acid in coral biomineralisation under ocean acidification scenarios. Corals cultured at 750 µatm pCO_2_ have higher skeletal [Asx] compared to their genotype analogues cultured at 180 µatm and 400 µatm (by 99% and 61% respectively). ECM Ω in the branching coral *Stylophora pistillata* cultured at 400 µatm and 25 °C is ~ 12^39^. At this Ω, raising seawater [aspartic acid] from 0.1 to 0.4 mM (our current best estimate of the ECM concentration in corals cultured at 180 and 750 µatm respectively) increases the biomolecule inhibition of aragonite precipitation from ~ 3 to ~ 11% (Fig. 5c). In reality, the ECM Ω is likely to be reduced at high seawater pCO_2_, as evidenced by reductions in pH_ECM_^4,21^ and coral calcification rate^2^. This Ω decrease will further exacerbate the inhibition of aragonite precipitation by aspartic acid (Fig. 5c). For the colonies analysed here, calcification at 750 µatm is reduced by 49% and 28% compared to the corals cultured at 180 µatm and 400 µatm pCO_2_ respectively^20^. We conclude that the inferred change in ECM [aspartic acid] may be responsible for a substantial proportion of this calcification decrease.

Our study demonstrates that coral calcification is affected by both the concentration of organic molecules at the calcification site, and the DIC chemistry in the ECM^21^. Further research on the response and interactions of ECM organic materials and DIC chemistry to increases in seawater pCO_2_ and temperature is vital to understanding the response of coral calcification to future climate change.

## Methods

### Coral culturing experiments

Massive *Porites* spp. corals were cultured at 25 °C and at 3 different seawater pCO_2_ conditions (180 µatm, 400 µatm and 750 µatm^20–22^). Heads were imported from Fiji, identified to species level based on corallite morphology and were considered to represent different genotypes when they were collected from spatially separate (non-adjoining) colonies. Heads were sawn into multiple pieces (each ~ 12 cm in diameter) so that at least one piece of each genotype could be cultured in each seawater pCO_2_ treatment. Corals were maintained at the different pCO_2_ treatments for 5 months, stained with Alizarin Red S to create a time horizon in the skeletons and then cultured for another 5 weeks before sacrifice. Corals were immersed in 3–4% sodium hypochlorite for 24–48 h with intermittent ultrasonic agitation to remove the tissues, then rinsed in distilled water. A slice was cut through the centre of each coral skeleton, parallel to the maximum growth axis using a water lubricated rock saw, cleaned in an ultrasonic bath and dried. Samples for amino acid analysis were obtained by drilling the skeleton deposited between the skeleton surface and the stain line i.e. representing the final 5 weeks of skeletal growth.

### Organic analysis

This method follows the organics analysis in Tomiak et al.^40^. To isolate the intracrystalline fraction of amino acids < 20 mg of skeletal sample was weighed in to a microcentrifuge tube and bleached using 50 μL 12% NaOCl per mg, the fraction was below 100 μm. The sample was re-agitated over 48 h before the bleach was removed. The remaining sample was rinsed five times by centrifuging with 18.2 MOhm milli-Q 200 μL MeOH was added to the Eppendorf and pipetted off after several minutes. Samples were dried overnight. To hydrolyse samples, < 10 mg was weighed in to a 2 mL sterile vial and 20 μL/mg 7 M HCl was added. Vials were flushed with nitrogen and placed in an oven at 110 °C, vial caps were then retightened to prevent drying. After 24 h, the samples were removed and dried in a centrifugal evaporator. Samples were rehydrated with 10 μL/mg rehydration fluid (0.01 M HCl + 1.5 mM NaN_3_, spiked with L-homo-arginine). Samples were analysed using reverse-phase HPLC with florescence detection, following the method of Kaufman and Manley^41^. This enables quantification of L and D isomers of 12 amino acids. Asparagine and glutamine undergo deamination during the preparation process and may therefore contribute to observed concentrations of aspartic acid and glutamic acid. However other methods e.g. proteomics, suggest that skeletal proteins are dominated by aspartic acid^24^ and we consider the asparagine contribution to be small. Blanks and standards were run throughout; all samples were ran in duplicate.

The precision of amino acid characterisation was estimated by splitting drilled powders before amino acid extraction and by drilling skeletal samples from different sections of the same coral head (Table S1). To estimate the impact of inadvertent inclusion of the Alizarin Red stain in the drilled sample, we compared the amino acid compositions of a sample drilled along the stain line and a powder drilled along a plane parallel to the stain and immediately above it. The amino acid concentrations agree within replicate precision for total, and each of the amino acids with the exceptions of serine and phenylalanine, which both occur in low levels in the skeletons (Table S1). Inclusion of the stain did not affect the mol% of different amino acid groups.

### In-vitro aragonite precipitation experiments

Synthetic aragonite was precipitated from seawater using a pH stat titrator (Metrohm Titrando 902) coupled with a gas system designed to produce air with a range of CO_2_ concentrations^20–22^. We adjusted the pH and [DIC] chemistry of the seawater and added amino acids to explore the impacts of these variables on precipitation. Precipitations were conducted over a pH_NBS_ of 8.34–8.69 (similar to that observed in the coral ECM, Fig. 1) and a [DIC]_seawater_ of 3,000–8,000 µmol kg^−1^. The coral [DIC]_ECM_ in *Stylophora pistillata* cultured at 400 µatm and 25 °C is ~ 3,000 µmol kg^−1^^39^.

Precipitations were conducted in natural seawater (salinity 35) collected from Crail, Fife, filtered (0.45 µm cellulose nitrate filter) and stored in a blacked-out 1000 L high density polyethylene (HDPE) container, under air with a CO_2_ atmosphere of ~ 400 µatm. For each precipitation, 320 mL seawater was decanted into a HDPE plastic beaker (total volume ~ 360 mL) capped with an ethylene tetrafluoroethylene lid with multiple ports and immersed in a water bath at 25 °C. A high precision combined pH/temperature sensor (Metrohm Aquatrode PT1000), a propeller stirrer, a gas tube and 2 titrant dosing tubes were inserted through the lid and into the headspace (gas tube) or seawater (all others). To avoid invasion or outgassing of CO_2_, the precipitating solution was maintained under a headspace with an air gas stream with pCO_2_ set to a value at equilibrium with the solution. Prior to the addition of the seed the gas stream was set to ~ 400 µatm CO_2_. At the point of seed addition and thereafter the gas stream [CO_2_] was altered to that in equilibrium with the seawater (calculated using CO_2_ sys v2.1 from seawater [DIC] and pH_NBS_ using the equilibrium constants for carbonic acid^42^, for KHSO_4_^43^ and for [B]^44^. For each precipitation the amino acid (if used) was added first by suspending the amino acid in 1 mL of seawater and then pipetting this into the reaction vessel. The pH of the seawater was adjusted back to the starting value with the addition of 1.0 M NaOH.[DIC] was increased with the addition of 0.6 M Na_2_CO_3_ and the pH adjusted to the required value by addition of 1.0 M HCl or NaOH. Once pH was stable 400 mg of an aragonite seed (from 2 batches of ground coral skeleton with surface areas of 3.45 and 4.41 m^2^ g^−1^ as analysed by the Brunauer–Emmett–Teller technique)^45^ was added to the reaction vessel to act as a surface for mineral growth. Precipitation of CaCO_3_ consumes DIC and Ca^2+^ and reduces solution pH. The associated pH decrease triggered the titrator to add equal volumes of 0.45 M Na_2_CO_3_ and 0.45 M CaCl_2_. The titration continued until 5 mLs of each titrant had been added resulting in the precipitation of ~ 225 mg of CaCO_3_. Seawater temperatures within and between precipitations varied by < 0.2 °C. XRD and/or Raman spectroscopy of a least one precipitate produced under each set of conditions confirmed that the original seeds and precipitates were aragonite. Post-precipitation the sensor, beaker and propeller stirrer were submerged in 0.1 M HCl to dissolve any precipitate and then rinsed thoroughly with deionized water.

The pH sensor was calibrated with NIST buffers daily and are reported on the NBS scale. Buffers were replaced each week and the maximum difference observed between old and fresh buffers in a single session was 0.004 pH units. Seawater [DIC] was measured at the start and end of a subset of experiments using an Apollo Sci Tech AS-C3 DIC analyser to ensure that seawater [DIC] was as expected and to monitor for any CO_2_ invasion or outgas during precipitations. Measured seawater [DIC] agreed with predicted seawater [DIC] within instrumental error^46^ and [DIC] variations within single precipitations were typically 3% and always < 15%. Seawater [Ca^2+^] was 10.1 mM as analysed by ICP-OES after 1:50 dilution with 3% HNO_3_ and spiking with 1 ppm Y as the internal standard. Seawater [Ca^2+^] was measured using a Ca^2+^ ion selective electrode (Metrohm) in filtered, acidified (pH 4) samples collected at the start and end of a subset of precipitations. Samples were maintained at constant temperature and measured consecutively to reduce electrode drift. Reproducibility of standards measured before and after samples was ≤ 5% and seawater [Ca^2+^] at the start and end of the precipitations always agreed within this value.

A sample titration is illustrated in Figure S4 indicating the tight control of pH during precipitation. The aragonite precipitation rate was estimated from the linear fit between time and titrant dosed and was normalized to the surface area of the starting seed. Control experiments, conducted under each set of precipitation conditions but without the addition of seed, demonstrated that no homogenous nucleation occurred in the solutions over the time scale of the precipitation i.e. no dosing of titrants occurred. Each set of precipitation conditions was tested multiple times (n = 2–10) with the exception of the precipitations at Ω = 35.4 which were not replicated. Data for individual precipitations are summarized in the Supplementary information (Table S5).

### Identification of amino acid concentrations for precipitation experiments

We precipitated an estimated 225 mg of aragonite from a seawater solution with 2 mM aspartic acid onto 400 mg of inorganic aragonite seed. We estimate that the aragonite precipitated in vitro incorporated [aspartic acid] of 8,554 pmol mg^−1^ (based on RP-HPLC analysis of the final precipitate and starting seed respectively ([aspartic acid] = 7,700 pmol mg^−1^ and 17 pmol mg^−1^ respectively). We estimate an aspartic acid_aragonite_/aspartic acid_seawater_ partition coefficient of ~ 0.004 (mmol L^−1^/mmol g^−1^) indicating that the incorporation of the amino acid in the aragonite has a minimal effect (< 0.5%) on seawater [aspartic acid]. We estimate an [aspartic acid] of the coral calcification media of ~ 0.1–0.4 mM to yield the coral skeletal [aspartic acid] observed in the corals analysed here.

## Supplementary information

Supplementary Information 1.

Supplementary Table S5.
