# Supplementary material for: The role of aspartic acid in reducing coral calcification under ocean acidification conditions
Source: Sci Rep. 2020 Jul 30;10:12797. doi: 10.1038/s41598-020-69556-0 (PMC7393068; doi:10.1038/s41598-020-69556-0)
Supplement: Supplementary file 1 — Supplementary Information 1. [file 41598_2020_69556_MOESM1_ESM.pdf]

The role of aspartic acid in reducing coral calcification under ocean acidification conditions

Kellock C, Cole C, Penkman K, Evans D, Kroger R, Hintz C, Hintz K, Finch A & Allison N

Supplementary tables and figures

Table S1. Concentrations of amino acids (pmol mg^{-1} skeleton) and contribution of each amino acid to total (mol %) in 3 coral genotypes (G1, G2 and G3) cultured at 25°C and over a range of seawater pCO_2 . We cultured and analysed 2 duplicate colonies of G3 at 400 and 750 µatm seawater pCO2. The precision of analyses was estimated from the pooled standard deviation (1σ) of extraction and analysis of splits of drilled powders and of skeletal samples drilled from different sections of the same coral head (n=5). To assess the effect of the Alizarin stain we compared the results of a sample drilled along a stain line with a sample drilled in the same plane immediately above it.

Genotype	pCO2 (µatm)	Asx	Gkx	Ser	L-Thr	L-His	Gly	L-Arg	Ala	Val	Phe	Leu	Ile	Total
**Skeletal samples (pmol mg^{-1})**														
	180	491	90	66	32	0	226	16	57	47	23	45	31	1025
G1	400	804	114	85	41	0	252	20	70	42	23	45	26	1429
	750	1024	157	78	33	0	256	13	81	59	30	59	38	1701
	180	627	111	88	47	49	160	22	62	48	29	54	31	1166
G2	400	1321	236	156	71	9	297	31	103	70	38	76	43	2285
	750	1070	182	139	74	61	193	34	96	69	36	72	42	1858
	180	562	102	86	51	44	162	22	59	48	28	54	31	1092
	400	647	118	80	51	42	181	12	66	60	30	67	43	1216
G3	400	582	104	87	49	51	164	23	61	47	27	54	30	1119
	750	880	187	119	69	60	243	38	85	62	41	79	41	1684
	750	1560	351	223	175	47	874	91	167	161	98	243	134	3602
**Contribution to total amino acid (mol %)**														
	180	46%	7%	6%	3%	0%	18%	1%	5%	4%	2%	4%	3%	-
G1	400	54%	8%	6%	2%	0%	15%	1%	5%	3%	2%	3%	2%	-
	750	59%	9%	6%	2%	0%	10%	0%	4%	4%	0%	3%	3%	-
	180	49%	9%	7%	4%	0%	13%	2%	5%	4%	2%	4%	2%	-
G2	400	54%	10%	6%	3%	0%	12%	1%	4%	3%	2%	3%	2%	-
	750	55%	9%	7%	4%	0%	7%	2%	5%	4%	2%	4%	2%	-
	180	52%	9%	5%	4%	0%	9%	0%	5%	5%	2%	5%	3%	-
	400	47%	8%	7%	4%	0%	14%	2%	5%	4%	2%	5%	3%	-
G3	400	47%	8%	7%	4%	0%	14%	2%	5%	4%	2%	4%	2%	-
	750	47%	10%	6%	3%	1%	13%	2%	5%	3%	2%	4%	2%	-
	750	38%	9%	5%	4%	1%	21%	2%	4%	4%	2%	6%	3%	-
**Analytical precision**														
Concentration (pmol mg^{-1})		34 (4%)	13 (7%)	12 (12%)	11 (18%)	5 (66%)	51 (20%)	7 (20%)	4 (5%)	10 (16%)	6 (18%)	18 (25%)	10 (24%)	125 (8%)
Mol % of all amino acids		2.1 (4%)	0.2 (2%)	0.3 (5%)	0.3 (8%)	0.2 (54%)	1.5 (10%)	0.2 (8%)	0.2 (3%)	0.4 (9%)	0.1 (7%)	0.5 (11%)	0.3 (13%)	-
Stained sample		1113	171	120	49	0	260	12	82	65	13	54	46	1873
Adjacent unstained sample		1024	157	78	33	0	256	13	81	59	30	59	38	1701

Asx = aspartic acid, Gkx = glutamic acid, Ser = serine, L-Thr = L-threonine, L-His = L-histidine, Gly = glycine, L-Arg = L-arginine, Ala = alanine, Val = valine, Phe = phenylalanine, Leu = leucine, Iso= isoleucine.

Table S2. Coefficients of determination (r^2) and p values for regressions between skeletal amino acid concentrations and seawater pCO_2, seawater pH and coral calcification rates (Cole et al., 2018). p≤0.05 are highlighted in bold.

	Seawater pCO_2		Seawater pH		Calcification rate	
Amino acid	r^2	p	r^2	p	r^2	p
Aspartic acid/asparagine	**0.48**	**0.019**	**0.48**	**0.018**	**0.54**	**0.010**
Glutamic acid/glutamine	**0.42**	**0.031**	**0.40**	**0.037**	**0.50**	**0.015**
Serine	0.30	0.083	0.28	0.091	**0.54**	**0.010**
L-Threonine	0.25	0.120	0.22	0.143	0.35	0.055
L-Histidine	0.05	0.503	0.03	0.583	0.14	0.260
Glycine	0.21	0.159	0.19	0.187	0.15	0.234
L-Arginine	0.25	0.118	0.21	0.156	0.30	0.081
Alanine	**0.41**	**0.034**	**0.39**	**0.041**	**0.46**	**0.022**
Valine	0.29	0.089	0.25	0.114	0.30	0.081
Phenylalanine	0.28	0.094	0.24	0.125	0.27	0.100
Leucine	0.25	0.118	0.22	0.148	0.24	0.130
Isoleucine	0.24	0.126	0.21	0.158	0.22	0.141
Total amino acid	**0.40**	**0.038**	**0.38**	**0.043**	**0.43**	**0.028**
ECM pH (Allison et al 2018)	**-**	**-**	**-**	**-**	**0.40**	**0.038**

Table S3. Intercept, gradients and goodness of fit of multiple linear regression models to predict coral calcification rates from skeletal amino acid concentrtaions and pH_{ECM}.

	Model excluding pH_{ECM}		Model including pH_{ECM}	
	Coefficients	p value	Coefficients	p value
Intercept	43.4	6.15	-214	0.018
[Total]	0.0658	0.018	0.0417	0.012
[Asx]	-0.0376	0.010	-0.0204	0.025
[Glx]	-0.0577	0.086	-0.0240	0.572
[Serine]	-0.0503	0.076	-0.101	0.047
[Alanine]	-1.085	0.377	-0.694	0.021
pH_{ECM}	-	-	29.6	0.0098

Table S4. Intercept, gradients and goodness of fit of a multiple linear regression model to predict inorganic aragonite precipitation rates from seawater pH, Ω and [aspartic acid].

	Coefficients	P-value
Intercept	2731	0.53
pH	-444	0.40
Ω	284	3.16×10^{-58}
[Aspartic acid]	-566	5.11×10^{-20}

Figure S1. Regressions between coral calcification rate (from Cole et al. 2018) and skeletal amino acid concentrations (this study) and reconstructed pHECM (from Allison et al., 2018). Asx = aspartic acid + asparagine, Glx = glutamic acid + glutamine.

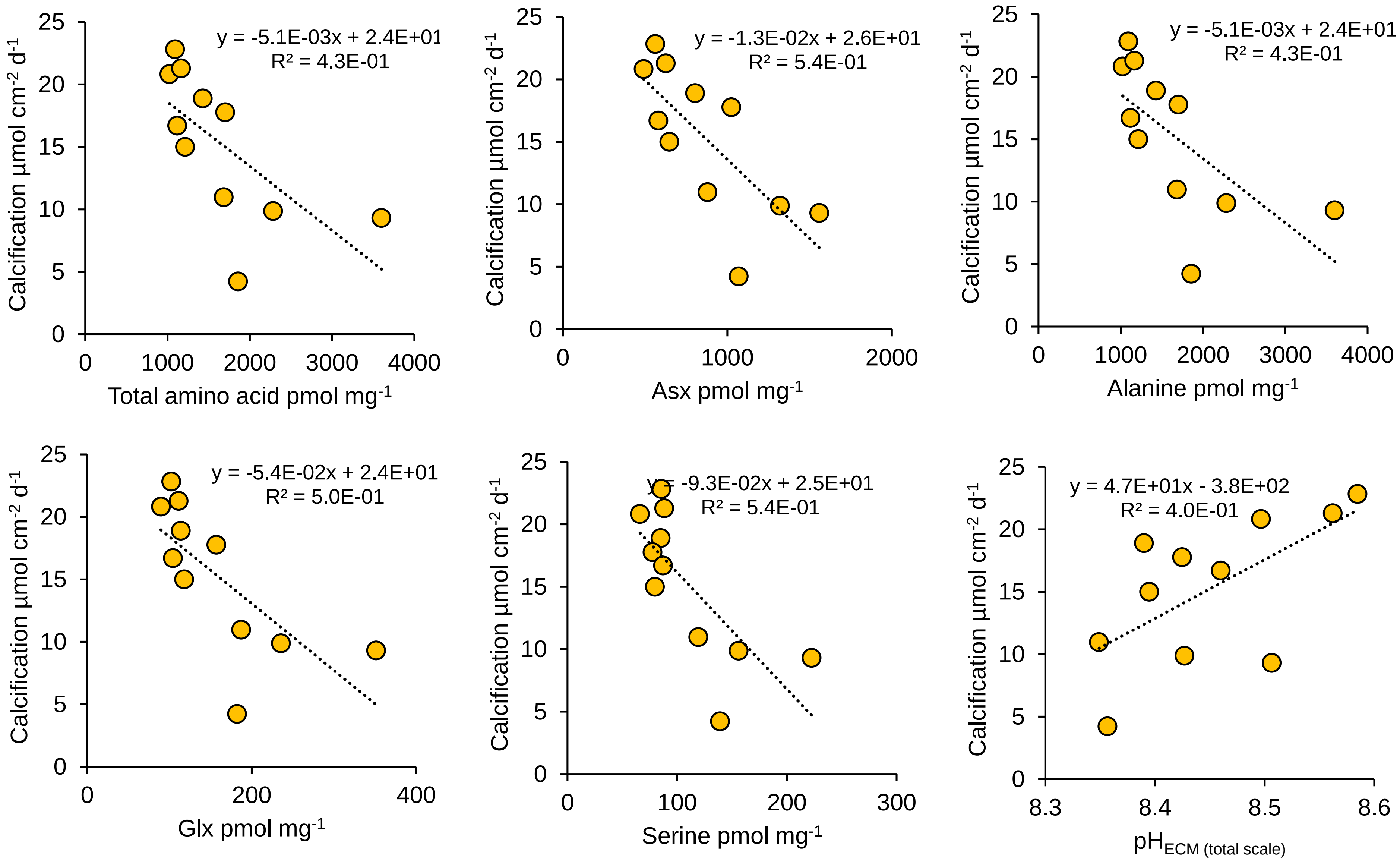

Figure S2. a) The total amount of skeletal amino acid produced by each colony each day (calculated from skeletal amino acid concentrations and coral calcification rates (Cole et al., 2018) and b) correlation between total skeletal amino acid produced per day and coral calcification rate.

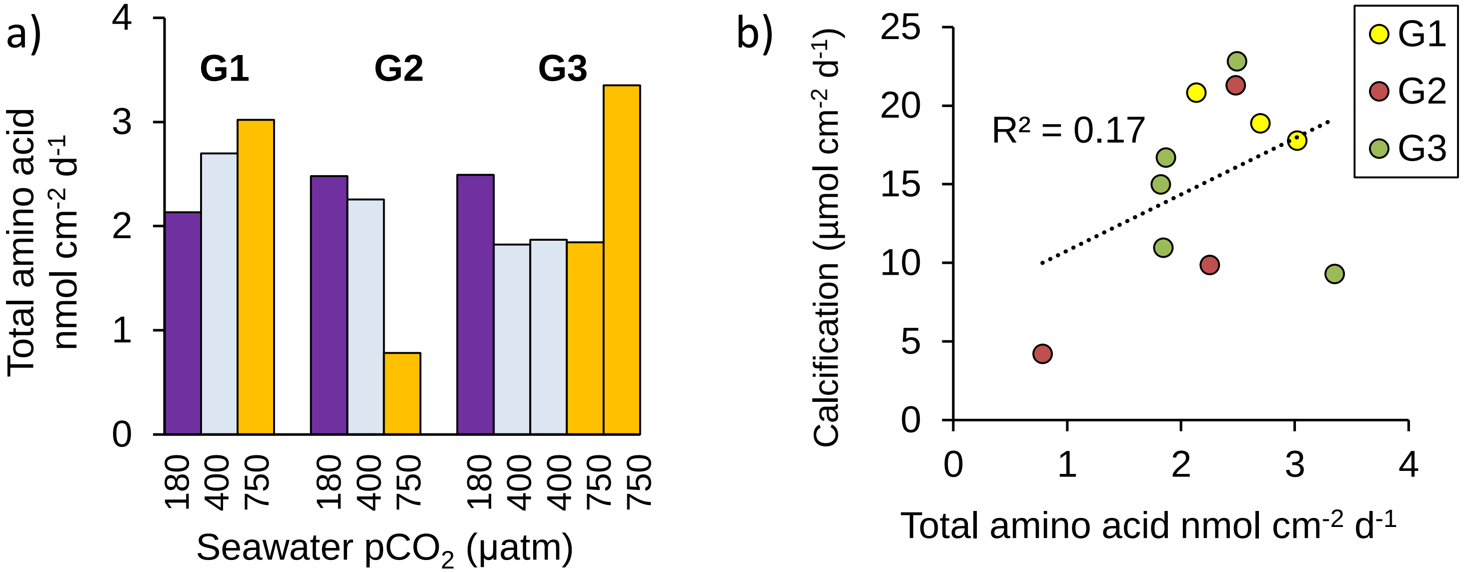

Figure S3. The percentage of total amino acids present as Asx (aspartic acid+asparagine) (mol %) correlated with percentage of a) glycine (p= 0.0024) and b) leucine (p=0.00060).

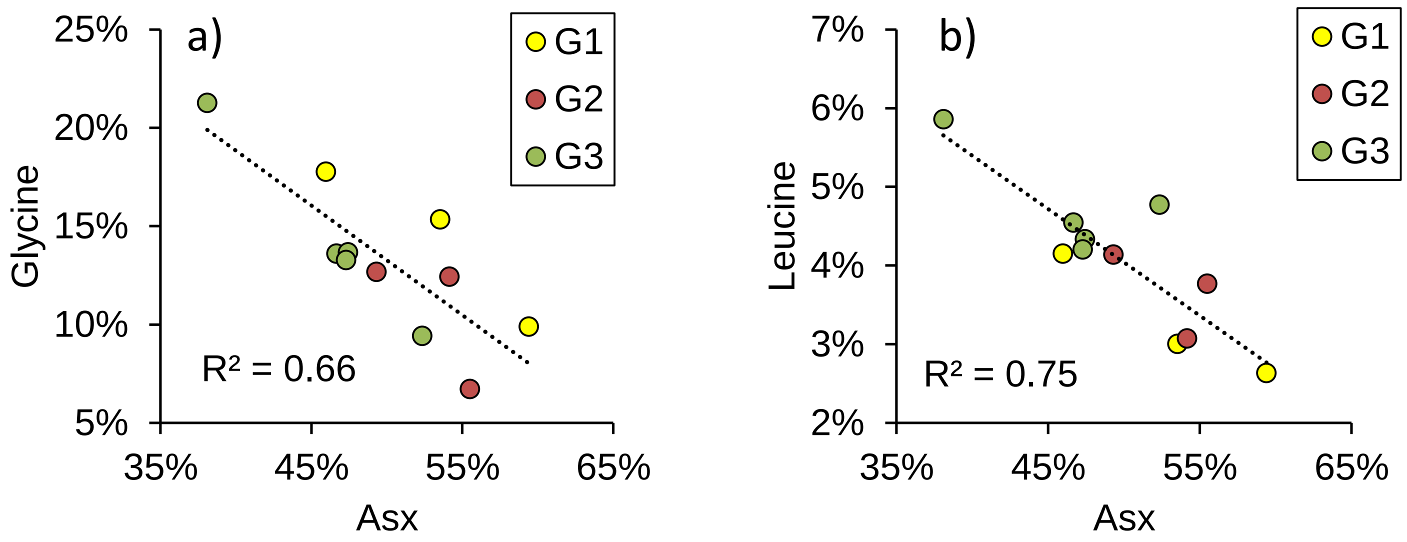

Figure S4. Profile of a typical titration showing control of seawater pH and linearity of titrant dosing. This experiment was conducted with pH 8.67, [DIC] = 2673, pCO2 = 142 µatm and Ω= 11.4,

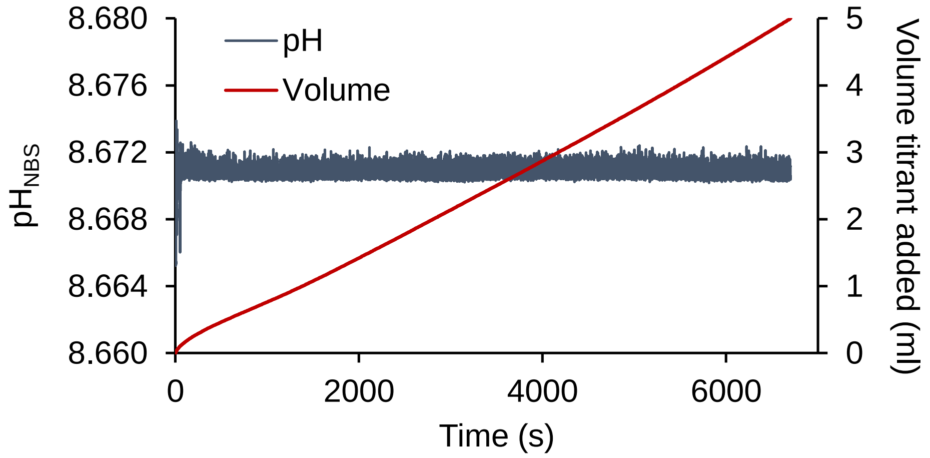

References: Cole, C., Finch, A. A., Hintz, C., Hintz, K. & Allison, N. Effects of seawater pCO2 and temperature on calcification and productivity in the coral genus Porites spp.: an exploration of potential interaction mechanisms. *Coral Reefs* **37**, 471–481 (2018).
